# EUS in Pediatrics: A Multicenter Experience and Review

**DOI:** 10.3389/fped.2021.709461

**Published:** 2021-08-25

**Authors:** Travis L. Piester, Quin Y. Liu

**Affiliations:** ^1^Keck School of Medicine of the University of Southern California, Children's Hospital Los Angeles, Los Angeles, CA, United States; ^2^Cedars-Sinai Medical Center, David Geffen School of Medicine at the University of California, Los Angeles, Los Angeles, CA, United States

**Keywords:** endoscopic ultrasonography, pediatric, pediatric gastroenterologists, pancreatitis, pancreatic fluid collection

## Abstract

**Background/Aim:** Endoscopic ultrasound (EUS) is a well-established tool used in the evaluation and treatment of a wide range of pathologies in adult medicine. EUS in pediatrics has been shown to be safe and technically effective, and its use continues to evolve. This article aims to describe the EUS experience at our tertiary-care centers with regard to safety, technical success, and its impact in clinical management. We also discuss the current and developing diagnostic and therapeutic uses for EUS in pediatrics such as in pancreaticobiliary disease, congenital anomalies, eosinophilic esophagitis, inflammatory bowel disease, and liver disease.

**Methods:** This is a retrospective review of EUS performed by two pediatric gastroenterologists trained as endosonographers between April 2017 and November 2020. Patient demographics, procedure indication, procedure characteristics, technical success, and complications were collected. Literature review was performed to describe current and future uses of EUS in pediatrics.

**Results:** Ninety-eight EUS were performed with 15 (15.3%) including fine needle aspiration/biopsy and 9 (9.2%) cases being therapeutic. Most common indications include choledocholithiasis (*n* = 31, 31.6%), pancreatic fluid collections (*n* = 18, 18.4%), chronic and acute recurrent pancreatitis (*n* = 14, 14.3%), and acute pancreatitis characterization (*n* = 13, 13.3%). Notable indications of pancreatic mass (*n* = 6, 6.1%) and luminal lesions/strictures (*n* = 6, 6.1%) were less common. Complications were limited with one instance of questionable GI bleeding after cystgastrostomy creation. Ninety-eight of 98 (100%) cases were technically successful.

**Conclusion/Discussion:** EUS has been shown to be performed safely and successfully in the pediatric population by pediatric endosonographers. This study and review support its use in pediatric practice and demonstrate the wide variety of indications for EUS such as pancreatic cystgastrostomy, celiac plexus neurolysis, and evaluation of chronic pancreatitis. This literature review also demonstrates areas of potential development for EUS within the practice of pediatric gastroenterology.

## Introduction

Endoscopic ultrasound (EUS) is a procedure that combines the direct intraluminal visualization of endoscopy with a sonographic exam of the GI tract and surrounding organs. This is most often accomplished with an echoendoscope, which has an ultrasound transducer built into the tip of a flexible endoscope. Since its introduction, EUS, followed by its combination with fine-needle aspiration/biopsy (FNA/B), has become a well-established tool in the evaluation and treatment of a wide range of pathologies in adult medicine (1, 2). Although it is used extensively in adult medicine, the use of EUS in pediatrics has been comparatively limited (3–9). Its use in pediatrics has been shown to be safe for patients >15 kg and continues to evolve (10–14). This article aims to describe the safe and successful use of EUS and its role in clinical management at two large academic referral centers. We will also discuss current and developing diagnostic and therapeutic uses for EUS in children.

EUS is performed commonly with two distinct types of echoendoscopes, a radial echoendoscope and curvilinear echoendoscope. The radial echoendoscope is used purely as a diagnostic tool with imaging produced in a 360° view, perpendicular to the scope. The curvilinear scope provides a ~120°-180° view parallel to the scope and is equipped with a working channel suitable for both diagnostic and interventional maneuvers, such as FNA/B or stent placement. The working channel is positioned so needles and devices can be visualized sonographically. The primary downside to the use of these echoendoscopes in pediatrics is their relatively large size in small children. These echoendoscopes are limited to patients who can accommodate their large-diameter and long transducer tip. Esophageal intubation of a small child with a standard EUS scope carries an increased risk of cervical esophageal perforation. Nevertheless, there are studies reporting successful EUS in children <1 year of age, with echoendoscopes usually safely utilized in children as small as 15 kg (4, 5, 12). In our experience, we have successfully performed therapeutic EUS in children as small as 12 kg.

EUS can also be performed using high-resolution miniprobes placed through standard endoscopes. These have higher frequencies, increasing their resolution but limiting their ability to examine deeper structures. These probes are thus well-suited to examine the mucosa or immediate vasculature of the GI tract but have limited view beyond this level and thus have limited use to evaluate surrounding anatomy such as the pancreas and biliary tract.

EUS has traditionally been most often used to evaluate pancreaticobiliary and GI lumen pathology in adult gastroenterology (3, 4, 6, 8, 9, 11, 15). In pediatrics, this continues to be the main indication for EUS (10). The benefits of using EUS for this pathology include the lack of ionizing radiation, the ability to combine with therapeutic/interventional procedures, and the dynamic and high-resolution images produced. EUS compares favorably to other imaging modalities such as CT and MRI for these reasons. Although its use in pediatrics is increasing, it is limited by patient size and the need for anesthesia. Additionally, there continues to be a dearth of training opportunities for pediatric gastroenterologists to learn this skill and thus a lack of skilled endosonographers still exists in many communities (8). Despite these limitations, our paper aims to discuss the safety, technical success, and clinical impact of EUS performed by pediatric gastroenterologists in our large patient experience, as well as the current and future role for EUS in children.

## Methods

After approval from the Institutional Review Boards of Children's Hospital Los Angeles (CHLA), Los Angeles, CA, USA, and Cedars Sinai Medical Center (CSMC), Los Angeles, CA, USA, we performed a retrospective review to identify all patients ≤ 18 years of age who underwent EUS and associated interventions between April 2017 and December 2020. The study was exempt from obtaining the consent due to its retrospective and chart review nature. Procedures were performed by one of two pediatric gastroenterology-trained endosonographers (TLP or QYL). EUS examinations were performed using the Olympus radial echoendoscope (GF-UE160), the Olympus curvilinear array echoendoscope (GF-UCT180 or GF-UC140P-AL5), or the Olympus miniprobe system (Olympus America, Inc., Center Valley, PA, USA). FNB was performed using the 22-G or 25-G Boston Acquire needle (Boston Scientific, Marlborough, MA, USA) or the SharkCore FNB Biopsy system (Medtronic, Minneapolis, MN, USA). FNA was performed using the 22-G or 25-G Boston Expect FNA needle (Boston Scientific, Marlborough, MA, USA), or the 19-G, 22-G, or 25-G Cook EchoTip FNA needle (Cook Medical, Indianapolis, IN, USA). Lumen apposing metal stent placement was performed using the AXIOS system (Boston Scientific, Marlborough, MA, USA). Although the majority of the procedures were performed under general anesthesia, some were performed under monitored anesthesia care. The type of anesthesia was deferred to the anesthesiologist, unless the EUS procedure was for pseudocyst drainage and cyst-gastrostomy creation in which general anesthesia was recommended and performed. Decision to perform EUS was made by the performing endosonographer based on the clinical management decision of each patient.

Patient demographics such as age, weight, and sex were collected. Also, procedure indication, procedure characteristics, technical success, and complications were collected. Procedures were defined to be diagnostic if the primary outcome of the procedure was for obtaining information used in diagnosis including the use of FNA/B. The procedure was defined as therapeutic if there was any associated intervention with the goal of treating or managing pathology. This includes cyst drainage, creation of a cystgastrostomy, or stent placement/removal.

We defined diagnostic success as the ability of EUS to sonographically evaluate the anatomy of interest (e.g., the bile duct or pancreas gland) or the ability to obtain diagnostic tissue by FNA/B. Therapeutic success was defined as the successful completion of the therapeutic maneuver as planned (e.g., creation of a cystgastrostomy). The EUS was defined to change management if the procedure directly leads to a treatment course (e.g., avoidance of ERCP, surgery, or chemotherapy), *via* either therapeutic maneuver or diagnostic information gained from the EUS.

Post procedure complication, as defined per ASGE guidelines, were recorded (16). Data were analyzed using descriptive statistics. Discussion of current practice was based on our findings, and wider/future indications were included to provide a more complete review of EUS in pediatrics.

## Results

From April 2017 through December 2020, 98 EUS procedures were performed on a total of 72 children, of which there were 34 males (42 procedures) and 38 females (56 procedures). Eighty-five cases (87%) were performed under general anesthesia with the remaining 13 cases (13%) performed under monitored anesthesia care. Patient age ranged 3–18 years with a mean age of 10.7 ± 4.5 years. Patient weight ranged 11.4–113 kg with a mean 49.9 ± 24.1 kg. Indications for the procedure were divided into pancreatic (*n* = 54, 55.1%), biliary (*n* = 34, 34.7%), luminal (*n* = 6, 6.1%), and other (*n* = 4, 4.1%) (Table 1). More specifically, the most common indications for EUS in this series were bile duct evaluation for choledocholithiasis (*n* = 31, 31.6%), pancreatitis (*n* = 27, 27.6%), pancreatic fluid collection (PFC) management (*n* = 18, 18.4%), and suspected pancreatic mass/biliary obstruction (*n* = 16, 16.3%). The majority of the EUS performed was diagnostic in nature (*n* = 89, 90.8%) with a minority (*n* = 9, 9.2%) being therapeutic (Table 2). Therapeutic cases represent the creation of cystgastrostomy and subsequent stent and PFC management. Seventeen of 98 procedures (17.3%) directly changed management. Nine cases ruled out the need for ERCP for choledocholithiasis. Four cases treated PFCs with the patient no longer requiring surgical intervention. Four cases made diagnoses that altered the expected clinical management (e.g., need for surgery or chemotherapy).

**Table 1:** Procedure indications.

**Indication category**	**Indication**	**Procedures, *n***	**% of total procedures**
Pancreatic		54	55.1%
	Pancreatic fluid collection	18	18.4%
	Acute pancreatitis	13	13.3%
	Chronic pancreatitis	8	8.2%
	Pancreatic mass	7	7.1%
	Acute recurrent pancreatitis	6	6.1%
	Autoimmune pancreatitis	6	6.1%
	Biliary obstruction of suspected pancreatic origin	3	3.1%
Biliary		34	34.7%
	Choledocholithiasis evaluation	31	31.6%
	Biliary stricture	5	5.1%
Luminal		6	6.1%
	Esophageal	5	5.1%
	Small bowel lesion	1	1.0%
Other		4	4.1%
	Abdominal pain	3	3.1%
	Liver evaluation	1	1.0%

**Table 2:** Procedural characteristics.

**Procedure characteristic**	**Procedure number, *n***	**Percentage of total**	**Notes**
Diagnostic	89	90.80%	
Therapeutic	9	9.20%	All related to cystgastrostomy and management
FNA/FNB performed	15	15.30%	Pancreatic mass−5
			Pancreatic fluid collection (PFC)−4
			Autoimmune pancreatitis−2
			Abdominal pain−1
			Liver biopsy−1
			Periampullary nodule−1
			Chronic pancreatitis−1
Changed management	17	17.3%	Avoid unnecessary ERCP−9
			Directly treat, to avoid surgery for PFC−4
			Obtain histology to determine management−4
Complications	1	1.0%	Possible GI bleed−1
Procedure successful	98	100.00%	

A complication was observed in one case. This was a suspected GI bleed after EUS with cystgastrostomy placement. It is unclear if this was related to the procedure as the patient had anemia but no overt signs of GI bleeding on repeat endoscopy or cross-sectional imaging. Overall, 98/98 (100%) of cases were deemed successful.

## Discussion

This series represents one of the largest studies on pediatric endosonography to date. We demonstrate that EUS is technically feasible and safe in the pediatric population, supporting previous case series. This study reflects the presence of pediatric gastroenterology-trained endosonographers within referral, academic practices, which may influence how EUS is used. In previous literature reviews, there is a variety of balances between diagnostic and therapeutic procedures, possibly reflecting varying accessibilities to an endosonographer for children (10, 11). In 2018, Bizzarri et al. (10) published a review of 19 articles describing a total of 634 EUS procedures in pediatrics. The Bizzarri review reflects differing practice patterns with several series being performed by adult gastroenterologists. Our series represents the highest concentration of patients reported to date (reporting 98 procedures in 3.75 years), reflecting a busy referral population, pediatric-trained endoscopists, and increasing use of EUS in pediatric centers. Compared to the Bizzarri review, our patient population was slightly younger (mean 10.7 vs. 12.7 years), with slightly more pancreaticobiliary indications (89.8 vs. 77.7%). Our series also showed similar use of FNA/B (15.3 vs. 15.5%). Since the Bizzarri review, there have been at least two series published on EUS in pediatrics. These series, despite having slightly different goals, continue to show similar indications and a positive clinical impact (17, 18).

EUS can impact the diagnosis and treatment course of pediatric diseases. Our series demonstrates the ability of EUS to change clinical management with diagnostic information that directly dictates treatment decisions or provide therapeutic interventions that avoid further surgical interventions in 17.3% of cases. This included cases of EUS ± FNA/B guiding treatment for congenital esophageal stricture and pancreatic pathology, EUS to exclude choledocholithiasis for unnecessary ERCP and intraoperative cholangiogram, and therapeutic EUS to manage PFC which avoids the need for external drains or surgical intervention (19).

Limitations of our study include its retrospective nature and lack of long-term follow up. Future goals would be to conduct long-term follow-up on these patients to better evaluate the impact of our series.

As illustrated in this series, EUS in pediatrics currently has the most use in the evaluation and treatment of pancreaticobiliary disorders. In addition, there are several other uses for EUS that have been used in pediatrics. The future of EUS in pediatrics will likely evolve from its current use in adult medicine as well as developing improvements to EUS such as elastography and contrast-enhanced EUS. These current and future uses of EUS in pediatrics warrant discussion here.

### Pancreaticobiliary

**Figure 1:**
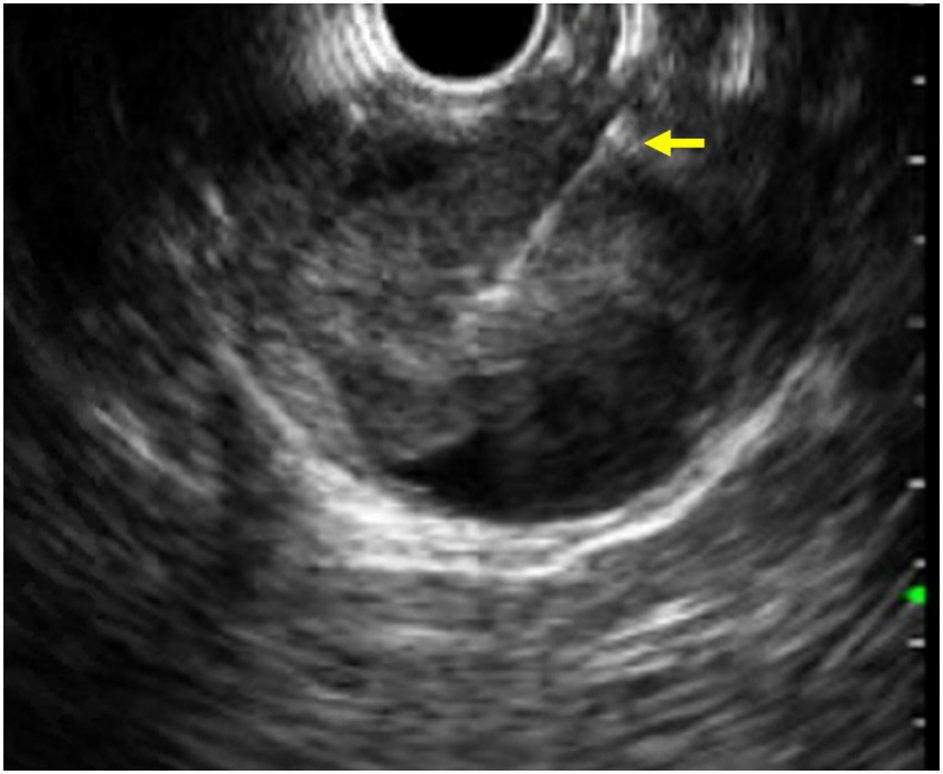
EUS image of a fine needle biopsy (yellow arrow) performed on a solid pseudopapillary tumor of the pancreas.

Use of EUS in pediatric pancreaticobiliary pathology includes the endosonographic evaluation and treatment of pancreatitis and PFC, the evaluation of the biliary tree most often to assess for choledocholithiasis, and the evaluation of pancreatic masses (Figure 1) (including autoimmune pancreatitis) which can present with biliary obstruction.

The incidence of pancreatitis in children is increasing (20, 21). EUS for pancreatitis is traditionally used in a diagnostic capacity to evaluate for potential etiologies for idiopathic acute recurrent pancreatitis (ARP) as well as to characterize changes associated with chronic pancreatitis (CP) (12). EUS has been shown to offer increased sensitivity for microlithiasis and gallstones that may explain ARP (22). Cross-sectional, non-invasive imaging has been used to evaluate for late findings of CP parenchymal changes such as pancreatic calcifications and dilated or obstructed pancreatic ducts (23). EUS offers the capacity to demonstrate more subtle changes in pancreatic parenchyma and ductal structures that are often not appreciated on non-invasive cross-sectional imaging or lab work (24–29). In the adult patient population, CP diagnosis with EUS is made by utilizing the Rosemont or conventional criteria which evaluate for changes such as parenchymal lobularity, hyperechoic foci/stranding, and ductal abnormalities (30–36). Although used in pediatrics, these criteria were derived utilizing adult patients, and to date, no validated EUS criteria exist for diagnosing CP in children. It should be noted that though these adult criteria are used in pediatrics, there are known age-related changes in the pancreas that can affect the sonographic appearance and it is well-described that pediatric CP has a much different etiology profile than adult cases (34, 37).

**Figure 2:**
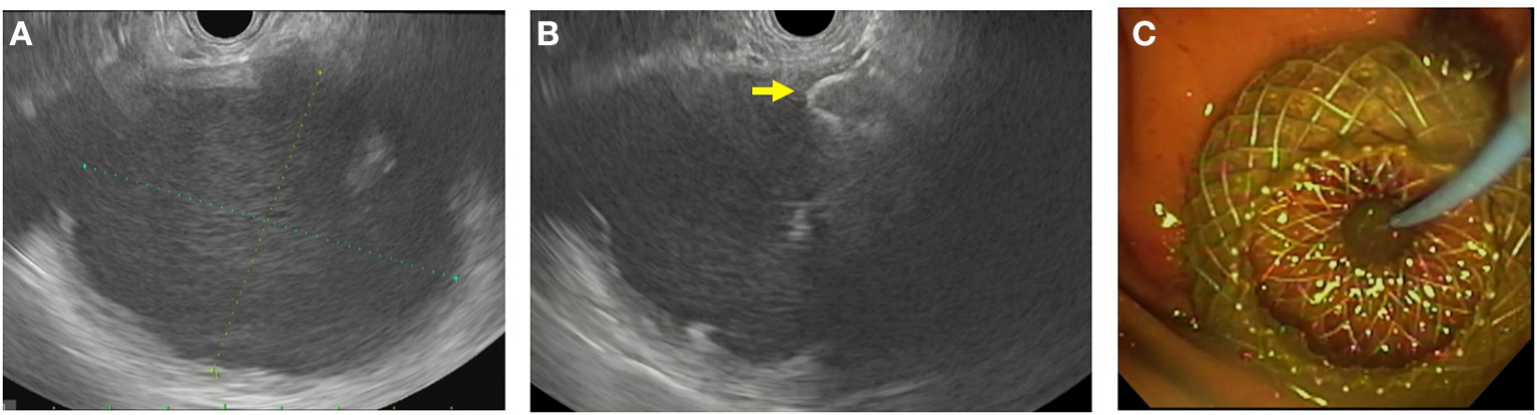
EUS-guided cystgastrostomy. **(A)** EUS image of a pancreatic pseudocyst. **(B)** Luminal apposing metal stent with a flange (yellow arrow) deployed in the pseudocyst. **(C)** Endoscopic image of the cystgastrostomy with the luminal apposing metal stent.

Much of pancreatic therapeutic EUS is for the management of PFC. These PFCs are often secondary to severe acute pancreatitis and categorized according to the revised Atlanta classification of 2012 (38). In cases where a symptomatic PFC has become mature enough, EUS-guided drainage and creation of cystgastrostomy/cystoduodenostomy can be considered (Figure 2) (39, 40). This is accomplished by using EUS with FNA to aspirate fluid from the fluid collection for cytology, fluid culture, and amylase levels. Cystgastrostomy and cystoduodenostomy were traditionally created *via* the Seldinger technique to ultimately place plastic stents from the lumen to the cyst. This has mostly been replaced with fully covered metal stents (FCMS), specifically the lumen apposing metal stent system, which places a large-bore, FCMS from the lumen to the cyst. Although commonly used in practice, the use of FCMS in pediatrics has been described but not widely studied (8, 41–44). In these cases, EUS visualization allows for vessel-free paths to be identified and confirmation of fluid characteristics. EUS can also be used therapeutically to perform celiac plexus block where an analgesic and steroid (or sclerosing agent) are injected at the celiac plexus. Unfortunately, this has shown limited benefit in adult patients with chronic pancreatitis and is also not widely studied in pediatrics (45, 46).

Pancreatic masses in children are rare but do occasionally present with signs and symptoms of biliary obstruction and/or vague symptoms such as abdominal pain. Like its use in adults, EUS for pancreatic lesions can evaluate the lesion size, location, and relationship to surrounding structures, which helps stage malignancies. Most importantly, EUS can aid in obtaining a tissue diagnosis using FNA/B. In addition to true pancreatic masses, autoimmune pancreatitis (AIP) can also present as a pancreatic mass (47). Tissue diagnosis is crucial for these patients to determine the correct treatment course and avoid erroneous surgical resection of an AIP lesion.

### Biliary Tree/Choledocholithiasis

**Figure 3:**
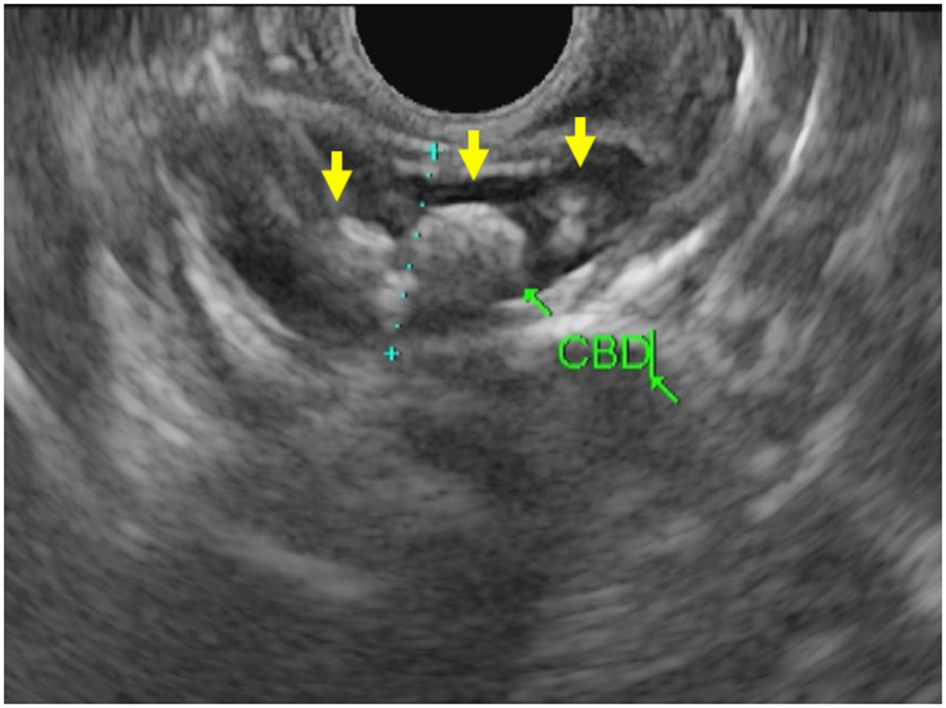
EUS images of the common bile duct with multiple choledocholithiasis (yellow arrows).

The biliary tree is visualized well with EUS. For this reason, EUS offers an excellent modality to assess pathology of both the bile duct itself and the surrounding structures such as the liver, pancreatic head, and porta hepatis. As reflected in our series, EUS in pediatrics is commonly used to evaluate the biliary tree for choledocholithiasis (Figure 3). EUS can be employed directly before ERCP to avoid performing unnecessary ERCP with its associated risks. In adult patients, there is a well-delineated role for imaging (MRCP or EUS) in cases with intermediate risk by labs, risk factors, and abdominal ultrasound (48). Unfortunately, the adult risk stratification has not been as predictive for children who would benefit from ERCP in these cases (49). In practice, EUS has shown excellent sensitivity and specificity for choledocholithiasis and thus can be valuable in settings with equivocal laboratory or MRCP results (50).

### Luminal EUS

EUS is well-suited to examine the gastrointestinal lumen because of the ability to differentiate between the five layers of the gastrointestinal wall: mucosa, muscularis mucosa, submucosa, muscularis propria, and serosa/adventitia. Because of this, it is often used in adults to stage GI malignancy and can be paired with FNA/B to help make tissue diagnosis during staging. In pediatrics, EUS can be used to evaluate luminal masses/lesions, but this is not as common as the adult population (51). EUS in pediatrics also has other uses. EUS can be used to evaluate congenital esophageal stenosis. This congenital malformation has three subtypes, and EUS is useful in determining if balloon dilatation is warranted or if surgical planning is needed (52). Eosinophilic esophagitis has been evaluated with EUS and shown to have significantly thicker portions of the luminal wall in two studies (53, 54). EUS has also been used in a variety of pathologies to evaluate the anorectal area. EUS can evaluate and treat varices, anal sphincter thickness/integrity, and postsurgical anatomy and monitor therapy in perianal IBD (55–59).

### Evolving and Future Use

EUS in pediatrics continues to evolve, following the path of EUS in the adult patient population. Similarly in adult patients, endosonographers are performing EUS-guided liver biopsy, varix therapy, and EUS-guided biliary access in children (60). As techniques become more common for adult patients, we can expect these procedures to be used and studied in pediatric patients. Also, on the horizon are contrast-enhanced EUS and EUS elastography, novel techniques that can improve the resolution and utility of the EUS exam (61–64). Contrast-enhanced EUS uses gas-filled microbubbles injected peripherally during the EUS exam. It has been used in adults to help differentiate pancreatic lesions (65, 66). Contrast-enhanced EUS shows information about vascularity and blood flow in a lesion and can be used to reveal or differentiate early necrotic foci and AIP from neoplasms (63). Elastography can be paired with the EUS to examine relative tissue stiffness and create a color map image. Early use of EUS elastography has been utilized in the evaluation pancreatic lesions and has been studied as a way to identify pancreatic fibrosis and predict risk of exocrine pancreatic insufficiency in CP (61, 62, 64, 67).

## Conclusion

In summary, this large case series illustrates how EUS is currently utilized in tertiary referral pediatric GI centers. The data highlight the diagnostic role for EUS in both pancreaticobiliary and luminal pathology. EUS can be both interventional and therapeutic and alter clinical management in children. Our series also shows that currently the most common indications for EUS in pediatrics is for pancreaticobiliary indications, and that safety and technical success are comparable with previous reported series. Further larger multicenter prospective studies can continue to elucidate the technical success, safety, and role of EUS in the clinical management of children.

## Data Availability Statement

The raw data supporting the conclusions of this article will be made available by the authors, without undue reservation.

## Ethics Statement

The studies involving human participants were reviewed and approved by IRB of Children's Hospital LA and Cedars-Sinai Medical Center. Written informed consent from the participants' legal guardian/next of kin was not required to participate in this study in accordance with the national legislation and the institutional requirements.

## Author Contributions

All authors listed have made a substantial, direct and intellectual contribution to the work, and approved it for publication.

## Conflict of Interest

The authors declare that the research was conducted in the absence of any commercial or financial relationships that could be construed as a potential conflict of interest.

## Publisher's Note

All claims expressed in this article are solely those of the authors and do not necessarily represent those of their affiliated organizations, or those of the publisher, the editors and the reviewers. Any product that may be evaluated in this article, or claim that may be made by its manufacturer, is not guaranteed or endorsed by the publisher.
